# Psychosocial Correlates of Insomnia Among College Students

**DOI:** 10.5888/pcd19.220060

**Published:** 2022-09-15

**Authors:** Yves Paul Vincent Mbous, Mona Nili, Rowida Mohamed, Nilanjana Dwibedi

**Affiliations:** 1 School of Pharmacy, Department of Pharmaceutical Systems and Policy, West Virginia University, Morgantown, West Virginia

## Abstract

**Introduction:**

Among college students, insomnia remains a topic of research focus, especially as it pertains to its correlates and the extent of its association with mental conditions. This study aimed to shed light on the chief predictors of insomnia among college students.

**Methods:**

A cross-sectional survey on a convenience sample of college students (aged ≥18 years) at 2 large midwestern universities was conducted from March 18 through August 23, 2019. All participants were administered validated screening instruments used to screen for insomnia, depression, and attention deficit hyperactivity disorder (ADHD). Insomnia correlates were identified by using multivariate logistic regression.

**Results:**

Overall, 26.4% of students experienced insomnia; 41.2% and 15.8% had depression and had ADHD symptoms, respectively. Students with depression (adjusted odds ratio, 9.54; 95% CI, 4.50–20.26) and students with ADHD (adjusted odds ratio, 3.48; 95% CI, 1.48–8.19) had significantly higher odds of insomnia. The odds of insomnia were also significantly higher among employed students (odds ratio, 2.10; 95% CI, 1.05–4.18).

**Conclusion:**

This study showed an association between insomnia and mental health conditions among college students. Policy efforts should be directed toward primary and secondary prevention programs that enforce sleep education interventions, particularly among employed college students and those with mental illnesses.

**Summary**

**What is already known on this topic?**

Despite the well-known prevalence of insomnia among college students, its association with mental health remains a topic of considerable interest, particularly among this vulnerable population constantly adapting to the demands of the academic world.

**What is added by this report?**

We show that at least a quarter of college students experience insomnia, and we uncover its predominant association with attention deficit hyperactivity disorder and depression.

**What are the implications for public health practice?**

The implications demand a serious consideration of mental health during attempts to improve students’ sleep quality.

## Introduction

The National Sleep Foundation and the American Academy of Sleep Medicine and Sleep Research Society guidelines recommend 7 to 9 hours of sleep for young adults (1). However, at least 60% of college students have poor quality sleep and garner, on average, 7 hours of sleep per night (2). Previous research showed that up to 75% of college students reported occasional sleep disturbances, while 15% reported overall poor sleep quality (3). In another work, among a sample of 191 undergraduate students, researchers found that 73% of students exhibited some form of sleep problem, with a higher frequency among women than men (4).

Direct consequences of poor sleep among college students include increased tension, irritability, depression, confusion, reduced life satisfaction, or poor academic performance (4). Evidence abounds of the positive correlation between academic failure, low grade point average, negative academic performance, and poor sleep quality patterns (5). As these complications arise early in the life of these students, they might develop into serious ailments as they grow older (high blood pressure, diabetes, stroke) and thereby create an even bigger public health problem. Because insomnia weakens physical and mental functions in addition to academic performances, reduced sleep quality could also lead to mental issues or vice versa (6).

Erratic schedules and lifestyle adjustments coupled with the strain of daily occupation are partly to blame for the general dissatisfaction with sleep quality and duration, because work obligations reduce hours of sleep among college students (2). However, in light of these consequences, it behooves the scientific community to identify modifiable factors associated with insomnia among college students that could help spur countermeasures or design lifestyle interventions to ameliorate the overall well-being of college students. In this study, we strived to identify environmental, mental, and behavioral factors affecting insomnia among college students. The intersection between behavioral factors and mental health is also evaluated in this work because physical activity, particularly, has been shown to mitigate insomnia (7). Because the relationship between insomnia and some of the understudied mental conditions could be bidirectional and given that cause-and-effect will not be established in this study, insomnia was labeled a criterion variable.

## Methods

### Study design, sampling, eligibility criteria

A cross-sectional design was used for this study. Convenience and snowball sampling strategy methods were used for sampling. West Virginia University and Marshall University students aged 18 years or older and able to read and write in English were eligible to participate. Study approval was acquired from the Institutional Review Board of West Virginia University. Consent for participation and anonymity were emphasized before the questionnaire’s distribution, along with instructions for completion. No incentives were provided for participants in this study.

### Instruments and measures

Demographic characteristics included sex (male, female), age, race (White; All others, which included Black or African American, American Indian or Alaska Native, Asian, Native Hawaiian or Pacific Islander, and any other racial group), marital status (married, not married), educational level (undergraduate, professional or graduate), employment status (employed, unemployed), physical activity (<2 d/wk, ≥2 d/wk), caffeine consumption (<6 cups/d, ≥6 cups/d, because previous research established a daily upper limit of 6 cups to maintain a healthy heart and blood pressure [8]), alcohol use (never, some days or every day), smoking status (yes, no), and the number of chronic non–mental health conditions (guided by the US Health and Human Services’ strategic framework [9], and included arthritis, asthma, cancer, chronic obstructive pulmonary disease, Crohn disease, cystic fibrosis, diabetes, epilepsy, heart disease, HIV/AIDS, and multiple sclerosis).

The criterion variable in this study was a diagnosis of insomnia as assessed by the Insomnia Severity Index (ISI). The ISI uses 7 items to evaluate the severity of insomnia. The first 3 items assess severity of sleep onset, sleep maintenance, and early morning awakening problems, and the last 4 examine sleep satisfaction, sleep disturbance, sleep worry, and sleep interference in daily life (10). Each item is graded on a 0 to 4 Likert scale, and the total score is calculated as the sum of each item, yielding minimum and maximum values of 0 and 28, respectively. Total score categories are as follows: 0 to 7 = no clinically significant insomnia; 8 to 14 = subthreshold insomnia; 15 to 21 = clinical insomnia (moderate severity); 22 to 28 = clinical insomnia (severe). In this study, ISI scores were divided into 2 categories based on a cutoff point of 15: patients with clinically significant insomnia (cutoff point of 15 or more) and participants with no clinically significant insomnia (cutoff point less than 15). This threshold point was motivated by the validity of this scale as a primary care diagnostic tool at a cutoff score of 14 (11).

Instruments to screen for depression and attention deficit hyperactivity disorder (ADHD) were used to evaluate mental health. For depression, we used the Patient Health Questionnaire (PHQ-9), a self-reported questionnaire that contains 9 items incorporating the Diagnostic and Statistical Manual of Mental Disorders (DSM) IV criteria for probable major depressive disorder. Each item can be scored from 0 through 3, and total scores can vary from 0 to 27, with cutoff points of 5, 10, 15, and 20, corresponding respectively to diagnoses of mild, moderate, moderately severe, and severe depressive symptoms. Given the high correlation observed in the literature between the third item of the PHQ-9 (also assessing sleep disturbance) and various sleep scales (12,13), we removed this item before calculating the overall score. PHQ-9 scores were divided into 2 categories: participants with clinically significant depressive symptoms (cutoff point of 8 or more) and participants with no clinically significant depressive symptoms (cutoff point less than 8). This was dictated by the sensitivity and specificity of the PHQ-9 at this cutoff score as a satisfactory diagnostic tool for depression in primary and secondary care settings (14).

For ADHD, Part A of the Adult ADHD Self-Report Scale (ASRS) was used. Only Part A of the questionnaire contains the 6 predictive measures of ADHD symptom severity (15). Items use a Likert scale (never, rarely, sometimes, often, very often). For items 1 to 3, ratings of sometimes, often, or very often were assigned 1 point (ratings of never or rarely were assigned 0 points). For the remaining items, ratings of often or very often were assigned 1 point (ratings of never, rarely, or sometimes were assigned 0 points). A sum of scores of 4 or more indicated ADHD symptoms. Diagnosis of anxiety was established using an item that elicited from participants a recent diagnosis of anxiety or current medication regimen for anxiety. The criterion variable and predictors in this study were collected using a 3-part questionnaire, including demographics, insomnia screening, and mental health screening.

### Survey procedure

The online survey was administered using the Qualtrics (Qualtrics) web-based survey tool. The invitation letter to participate in this survey was sent to participants through the listserve to students and social media outlets (Facebook and Twitter) from March 18 through August 23, 2019.

### Data analysis

During the analysis, we omitted responses with half or more missing information (75 incomplete and missing responses were excluded from the final sample) from the criterion variable (insomnia) and predictors (ie, ADHD, anxiety, depression, chronic non–mental health conditions, employment status, sex, race and ethnicity, sex, education level, physical activity status, alcohol and caffeine consumption, and smoking). Descriptive statistics were used to describe the study participants. Cell sizes with fewer than 5 were conflated with the next immediate encompassing category. Significant differences in outcomes among predictive factors were determined by using independent *t* tests. Differences were labeled significant at an α level less than or equal to .05. Were used χ^2^ tests of independence to compare the distribution of dependent categorical or nominal variables and the distribution in the criterion variable (for large cell sizes). Fisher tests were used for the same purpose, albeit for smaller cell sizes (~ n = 5). We did not apply any statistical adjustments (eg, Bonferroni adjustments) for multiple comparisons on the same sample out of concern for the substantial reduction in the statistical power of rejecting an incorrect Ho in each test (16).

Multivariable logistic regression models were built to model a relationship between predictors and insomnia. We included logistic regression models analyzing the interaction between different mental conditions and between physical activity and mental health (diagnosis of anxiety, depression, or ADHD). Model 1 regressed the dependent variable on all independent variables. Models 2 through 4 added 2-way interactions between mental conditions, namely anxiety, ADHD, and depression, respectively, and physical activity. From each of these models, odds ratios were derived. The analysis was conducted by using SPSS version 26 (IBM Corp).

### Validity and reliability

To validate the use of the foregoing instruments in a college population, we conducted confirmatory factor analyses. Results indicated loading patterns consistent with the structure of the adopted scales. Our method of choice was principal component analysis with varimax rotation. The ISI was a unidimensional scale with factor loading ranging from 0.375 to 0.876. The unidimensional PHQ-9 factor loadings oscillated between 0.627 and 0.881. The ASRS, also unidimensional, had factor loadings ranging from 0.462 to 0.803. The reliability of the ISI, PHQ-9, and ASRS, as assessed using the Cronbach α (0.857, 0.909, 0.768, respectively), was excellent. The degree of concordance between the ISI and the nonsleep scales (divergent validity) was evaluated by using correlation coefficients. We found a weak to moderate magnitude of correlation (*r* < 0.7), based on a widespread threshold from the literature (17).

## Results

A total of 330 responses were included in our analysis (Table 1). The mean age of participants was 24.4 years old. Across the entire sample, most participants were women (67.0%), White (89.7%), not married (94.2%), undergraduate students (62.4%), and with no chronic non–mental health conditions (69.7%). Based on the screening questionnaires, the prevalences of anxiety, depression, ADHD, and insomnia were 28.5%, 41.2.%, 15.8%, and 26.4%, respectively.

**Table 1:** Descriptive Statistics of Predictive Factors and Criterion Variable Among College Students, 2 Midwestern Universities, 2019^a^

Variable	Total	No Insomnia	Insomnia	*P* value
**Total**	**330 (100)**	**243 (73.6)**	**87 (26.4)**	** NA**
**Age, mean (SD), y**	24.4 (4.4)	25.1 (4.6)	23.7 (3.7)	.084^b^
**Sex**				
Male	109 (33.0)	93 (38.3)	16 (18.4)	.001^c^^,^ d
Female	221 (67.0)	150 (61.7)	71 (81.6)	
**Race**				
White	296 (89.7)	223 (91.8)	73 (83.9)	.04^c^^,^ e
All others^f^	34 (10.3)	20 (8.2)	14 (16.1)	
**Marital status**				
Not married	311 (94.2)	226 (93.0)	85 (97.7)	.29^g^
Married	19 (5.8)	17 (7.0)	2 (2.3)	
**Level of education**				
Undergraduate	206 (62.4)	149 (61.3)	57 (65.5)	.49^c^
Professional or graduate	124 (37.6)	94 (38.7)	30 (34.5)	
**Employed**				
No	178 (53.9)	132 (54.3)	46 (52.9)	.82
Yes	152 (46.1)	111 (45.7)	41 (47.1)	
**Physical activity**				
<2 d/wk	48 (14.5)	30 (12.3)	18 (20.7)	.06^c^
≥2 d/wk	282 (85.5)	213 (87.7)	69 (79.3)	
**Caffeine consumption**				
<6 cups/d	298 (90.3)	221 (90.9)	77 (88.5)	.56^g^
≥6 cups/d	20 (6.1)	16 (6.6)	4 (4.6)	
**Alcohol use**				
Not at all	116 (35.2)	88 (36.2)	28 (32.2)	.19
Some days or every day	214 (64.8)	155 (63.8)	59 (67.8)	
**Smoking status**				
Not at all	310 (93.9)	229 (94.2)	81 (93.1)	.49^g^
Every day and some days	20 (6.1)	14 (5.8)	6 (6.9)	
**Chronic non–mental health conditions**				
0	230 (69.7)	179 (73.7)	51 (58.6)	<.001^c^^,^ h
1	72 (21.8)	52 (21.4)	20 (23.0)	
≥2	28 (8.5)	12 (4.9)	16 (18.4)	
**Anxiety**				
No	236 (71.5)	181 (74.5)	55 (63.2)	.05^c^^,^ e
Yes	94 (28.5)	62 (25.5)	32 (36.8)	
**Depression**				
No	192 (58.2)	173 (71.2)	19 (21.8)	<.001^c^^,^ h
Yes	136 (41.2)	68 (28.0)	68 (78.2)	
**ADHD**				
No	276 (83.6)	222 (91.4)	54 (62.1)	<.001^c^^,^ h
Yes	52 (15.8)	21 (8.6)	31 (35.6)	
**Depression and ADHD**				
No	286 (86.7)	227 (93.4)	59 (67.8)	< .001^c^^,^ h
Yes	40 (12.1)	14 (5.8)	26 (29.9)	
**Depression and anxiety**				
No	280 (84.8)	217 (89.3)	63 (72.4)	<.001^c^^,^ h
Yes	48 (14.5)	24 (9.9)	24 (27.6)	
**Anxiety and ADHD**				
No	314 (95.2)	239 (98.4)	75 (86.2)	.001^c^^,^ d
Yes	14 (4.2)	4 (1.6)	10 (11.5)	

Abbreviations: ADHD, attention deficit hyperactivity disorder; NA, not applicable.

a Data are number (percentage) unless otherwise specified. Numbers may not add to total because of missing data.

b Independent *t* test.

c Pearson χ^2^.

d *P* value between .001 and <.01.

e *P* value between .01 and <.05.

f All other races included Black or African American, American Indian or Alaska Native, Asian, Native Hawaiian or Pacific Islander, and any other racial group.

g Fisher exact test.

h *P* < .001.

Among the participants with insomnia, most were women (81.6%), White (83.9%), undergraduate students (65.5%), physically active on 2 or more days during the week (79.3%), consumed less than 6 cups of caffeine per day (88.5%), at least occasionally consumed alcohol (67.8%), were nonsmokers (93.1%), had no chronic conditions (58.6%), were not anxious constantly (63.2%), were depressed (78.2%), and had no symptoms of ADHD (62.1%). In general, participants without insomnia followed the same trend, except that most did not have depression (71.2%). Employment status in both groups (participants with and those without insomnia) was roughly similar. Sex, race, the number of chronic non–mental health conditions, depression, and ADHD symptoms were found to be significant correlates of insomnia (Table 1).

Findings from models 2 and 4 were not significant. In model 3, the multiple logistic regression model indicated that psychosocial factors such as employment status, depression, and ADHD significantly increased the odds of insomnia (Table 2). Employed students had 2.10 times higher odds of insomnia compared with unemployed students. In addition, the odds of insomnia were 9.54 and 3.48 times higher for students with depression and ADHD, respectively. Anxiety was not significantly associated with insomnia (adjusted odds ratio: 1.71, *P* = .13). Physical activity was a significant effect modifier in the association between ADHD and insomnia (adjusted odds ratio: 12.1, *P* = 0.012). The strength of the association between ADHD symptoms and insomnia was lower among students who exercised 2 or more days a week compared with those who exercised less.

**Table 2:** Adjusted Association of Predictors With Insomnia, 2 Midwestern Universities, 2019

Predictor	Adjusted Odds Ratio (95% CI)	*P* value
**Age, y**	1.07 (0.95–1.20)	.26
**Sex**		
Male	1 [Reference]	.09
Female	1.93 (0.90–4.17)	
**Race**		
All others^a^	1 [Reference]	.35
White	0.61 (0.21–1.73)	
**Marital status**		
Not married	1 [Reference]	.19
Married	1.51 (0.82–2.80)	
**Level of education**		
Undergraduate	1 [Reference]	.96
Professional or graduate	0.98 (0.39–2.48)	
**Employment**		
No	1 [Reference]	.04^b^
Yes	2.10 (1.05–4.18)	
**Physical activity**		
<2 d/wk	1 [Reference]	.88
≥2 d/wk	0.94 (0.38–2.31)	
**Caffeine consumption**		
<6 cups/d	1 [Reference]	.17
≥6 cups/d	0.33 (0.07–1.60)	
**Alcohol use**		
Never or some days	1 [Reference]	.87
Every day	0.81 (0.07–9.54)	
**Smoking status**		
No	1 [Reference]	.36
Yes	1.55 (0.61–3.96)	
**Number of chronic non–mental health conditions**	1.65 (1.08–2.52)	.02^b^
**Anxiety**		
No	1 [Reference]	.13
Yes	1.71 (0.85–3.42)	
**Depression**		
No	1 [Reference]	<.001^c^
Yes	9.54 (4.50–20.26)	
**Attention deficit hyperactivity disorder**		
No	1 [Reference]	.004^d^
Yes	3.48 (1.48–8.19)	

a All other races included Black or African American, American Indian or Alaska Native, Asian, Native Hawaiian or Pacific Islander, and any other racial group.

b *P* value between .01 and <.05.

c *P* < .001.

d *P* value between .001 and <.01.

## Discussion

In this study, we identified factors associated with insomnia among college students. ADHD, depression, and employment status were significantly associated with insomnia. We reported a 26.4% prevalence of insomnia among college students, a finding consistent with existing literature. A previous meta-analysis reported an overall insomnia prevalence of 18.5% (95% CI, 11.2%–28.8%) among university students; our estimate fell within this reported CI (6). Another study found that insomnia prevalence was 26.7% among university nursing students (18). Taylor and coworkers reported an insomnia prevalence of 9.5% among a cohort of 1,039 college students by using the ISI and the Pittsburgh Sleep Quality Index (PSQI) (19); their operational definition of chronic insomnia was established over 3 months as opposed to 1 month in our study. In our work, small cell sizes restricted the categorization of insomnia into moderate, mild, or severe. This explains the deviation of our results from those of past researchers that used the ISI systematic classification of different degrees of insomnia. For instance, Gress-Smith et al found that 47% of college students had mild insomnia and 22.5% had moderate to severe insomnia (20). In another ISI-based study, 12% of students endorsed a diagnosis of clinical insomnia, and 45% met the criteria for subclinical insomnia (21). All these intricacies cement our results within the current pool of research.

Our findings indicated that 78.2% of students with insomnia also experienced depression, and the odds of insomnia were 9.54 times higher among students with depression than students without depression. Olufsen et al reported a prevalence of depression among college students with insomnia of 30% to 38%, using the Diagnostic and Statistical Manual of Mental Disorders, fourth edition (DSM-IV) (22). Another research concluded that depressive symptoms, assessed using criteria of the DSM-IV, were associated with increased insomnia complaints among college students (odds ratio, 1.09) (23). These findings lend credence to the bidirectional relationship between insomnia and depression. Thus, it is typical of patients with insomnia to exhibit psychological profiles (poor coping skills, poor health status, ruminative traits) that herald the onset of depression. Ubiquitous characteristics of insomnia, such as fatigue, irritability, and cognitive impairment, which are well-known derivatives of insomnia among students, exacerbate depressive symptoms (24).

In our sample, 15.8% had ADHD, and the odds of insomnia were 3.48 times higher for students with ADHD than those without ADHD. The prevalence of clinically significant cases of ADHD varies between 2% and 8% of the college student population (25). A previous study showed a similar ADHD prevalence to ours at ~19% (26). In the same study, the authors also reported that students with ADHD had a risk of insomnia 2.7 times greater than those without ADHD (26). These observations indicate the importance of examining symptom clusters that involve both sleep and mental and emotional components when investigating and treating insomnia, depression, or ADHD.

Physical activity mitigated the effect of mental health on insomnia. As regular physical activity helps improve sleep quality (7) and has psychological benefits (27), it was not surprising to find that among those with mental conditions, those who exercised more often (in this case, 2 or more days per week) seemed to have better sleep quality than those who exercised less. Students are often hesitant to seek help for mental health and insomnia concerns; therefore, interventions need to be youth-friendly, acceptable, feasible, and nonstigmatizing (28). Young people view physical activity as helpful in mitigating mental conditions as well as being nonstigmatizing (29). Although most university campuses offer physical activity–based wellness programs, research exploring students’ perceptions of on-campus physical activity initiatives as alternatives to mental health and insomnia management strategy is limited (30).

We found that employment was significantly associated with sleep problems among college students. Similarly, previous research has linked employment to insomnia. A meta-analysis found job demand to be negatively correlated with sleep quality, whereas job control was positively correlated (31). Students, most of whom held part-time jobs and thus had less job control yet high job demands, might understandably experience substantial sleep difficulties and reduced sleep quality in general. Also, the competing demands to complete academic requirements and maintain employment may also serve as structural barriers to adequate sleep.

### Strengths and limitations

This study had several strengths. First, we evaluated factors susceptible to accompany a diagnosis of insomnia in a sample of college students. Further, we used established instruments that we validated psychometrically across a new population. However, this study had a few limitations. First, the data were collected from 2 universities, namely West Virginia and Marshall University, thus limiting the generalizability of the findings. Information on study majors was not collected, yet could have influenced the prevalence and the uncovered associations of insomnia and mental conditions. Further, we used a cross-sectional design and could only establish association, not causality. Finally, small cell sizes restricted the stratification of insomnia, which would have enriched our results.

Our results indicate that better mental health and insomnia must be addressed concomitantly as their association is not random. Addressing these issues entails better time management skills dedicated to studying, work, and leisure. Such skills should be at the fingertips of college students to help them cope with the increasing demands of university life. These findings should also be communicated to the employers of college students who in turn should prioritize the overall well-being of their employees. As a future direction for our work, we endeavor to measure health services utilization among students with mental conditions that tie directly to sleep quality; this, in a bid, to inform policy on the need to improve mental health services access for college students.

### Conclusion

The burden of insomnia among college students is one that must be readily addressed as its spillover effects decrease substantial traits that are crucial for college life. Mental health, specifically depression and ADHD, and employment are salient contributors to the high levels of insomnia. Addressing these associations could help improve the experience and well-being of college students. Further, the promotion on campuses of healthy behaviors such as physical activity could yield significant improvements vis-à-vis the lifestyle of college students, as physical activity, in this study, has been shown to mitigate the effect of mental health on insomnia or vice versa.
